# Structural Dynamics Associated with Intermediate Formation in an Archetypal Conformational Disease

**DOI:** 10.1016/j.str.2012.01.012

**Published:** 2012-03-07

**Authors:** Mun Peak Nyon, Lakshmi Segu, Lisa D. Cabrita, Géraldine R. Lévy, John Kirkpatrick, Benoit D. Roussel, Anathe O.M. Patschull, Tracey E. Barrett, Ugo I. Ekeowa, Richard Kerr, Christopher A. Waudby, Noor Kalsheker, Marian Hill, Konstantinos Thalassinos, David A. Lomas, John Christodoulou, Bibek Gooptu

**Affiliations:** 1 Institute of Structural and Molecular Biology (ISMB), Department of Biological Sciences, Birkbeck College, London, WC1E 7HX, UK; 2 ISMB, Division of Biosciences, University College London, London, WC1E 6BT, UK; 3 Department of Medicine, University of Cambridge, Cambridge Institute for Medical Research, Cambridge, CB2 0XY, UK; 4 Division of Clinical Chemistry, Queen's Medical Centre, Nottingham, NG7 2UH, UK

## Abstract

In conformational diseases, native protein conformers convert to pathological intermediates that polymerize. Structural characterization of these key intermediates is challenging. They are unstable and minimally populated in dynamic equilibria that may be perturbed by many analytical techniques. We have characterized a *forme fruste* deficiency variant of α_1_-antitrypsin (Lys154Asn) that forms polymers recapitulating the conformer-specific neo-epitope observed in polymers that form in vivo. Lys154Asn α_1_-antitrypsin populates an intermediate ensemble along the polymerization pathway at physiological temperatures. Nuclear magnetic resonance spectroscopy was used to report the structural and dynamic changes associated with this. Our data highlight an interaction network likely to regulate conformational change and do not support the recent contention that the disease-relevant intermediate is substantially unfolded. Conformational disease intermediates may best be defined using powerful but minimally perturbing techniques, mild disease mutants, and physiological conditions.

## Highlights

► An α1-antitrypsin deficiency mutant is a *forme fruste* model for the Z variant ► NMR spectroscopic and ion-mobility mass spectrometric characterization of the mutant ► Residue-specific discrimination of disease-relevant and denaturant-induced ensembles ► A “clasp” motif caps a network of stabilizing interactions in α1-antitrypsin

## Introduction

**Figure 1:**
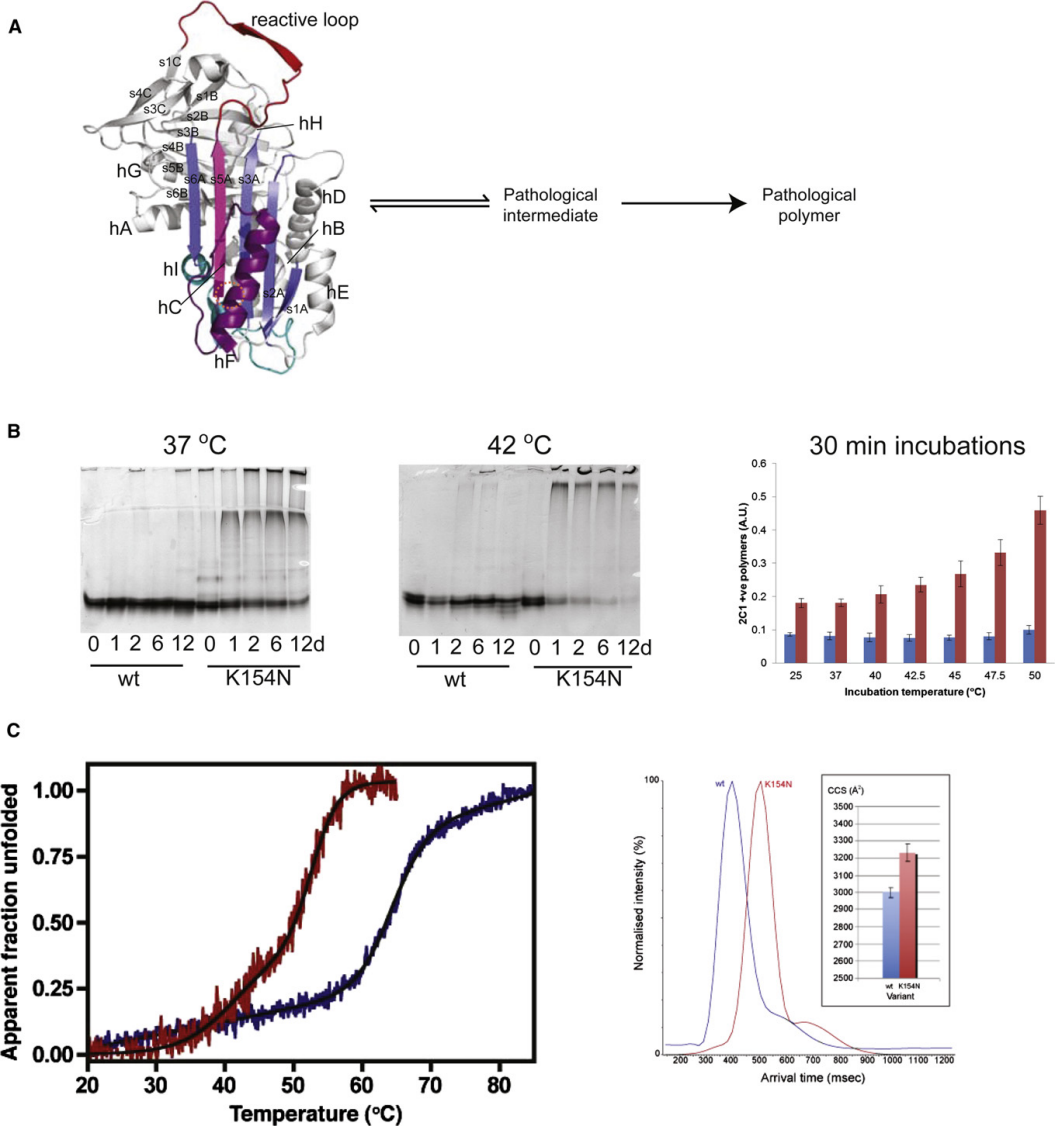
Pathological Polymerization of Lys154Asn α_1_-Antitrypsin (A) Polymerisation pathway from native conformer (mutation site circled). (B) 7.5% (w/v) native PAGE; polymerization of wild-type and Lys154Asn α_1_-antitrypsin (0.5 mg/ml protein [pH 7.4]) in vitro over 12 days at 37°C and 42°C. Polymerization is reported by loss of the monomeric band and the appearance of aggregated protein. (Right) Polymers formed by 30 min incubation between 40°C and 50°C of nonglycosylated wild-type (blue) and Lys154Asn (red) α_1_-antitrypsin are detected by 2C1 mAb ELISA (Z α_1_-antitrypsin polymer calibrated; Miranda et al., 2010). Differences between wild-type and mutant are significant (p < 0.05 to p < 0.005) for all temperatures. The increase in signal relative to starting material reaches significance for Lys154Asn α_1_-antitrypsin for incubations at 47.5 (p < 0.05) and 50 (p < 0.005)°C. Data are mean ± standard deviation (error bars) of three experiments. (C) (Left) Thermal denaturation CD spectroscopy (left; mean ellipticity at 222 nm, n = 10) for wild-type (blue) and Lys154Asn (red) α_1_-antitrypsin. (Right) Arrival times and collision cross-section (CCS) values calculated by IM-MS for wild-type (blue) and Lys154Asn α_1_-antitrypsin indicated a 7.8% increase in CCS in Lys154Asn relative to wild-type α_1_-antitrypsin at 39°C (no difference at 20°C and 34°C). Data are mean ± standard deviation (error bars) of three experiments. See also Figure S1.

The conformational diseases are characterized by conformational transitions in mutant proteins that allow multimerization and aberrant protein deposition. They include Alzheimer's disease and Lewy body dementia, the prion encephalopathies, systemic amyloidosis, and the serpinopathies (Lomas and Carrell, 2002). The serpinopathies result from the polymerization of mutants of members of the serine protease inhibitor or serpin superfamily of proteins (Gooptu and Lomas, 2009). The archetype of the serpinopathies is α_1_-antitrypsin deficiency. α_1_-antitrypsin is synthesized by hepatocytes (Laurell and Jeppsson, 1975) and released into the circulation, where it protects the lung from the actions of neutrophil elastase (Gooptu et al., 2009a). Severe α_1_-antitrypsin deficiency is found in 1:2000 individuals of North European descent (Blanco et al., 2001) and typically results from homozygosity for the Z allele (Glu342Lys). The Z mutation causes α_1_-antitrypsin to adopt an intermediate conformation and rapidly polymerize (Figure 1A; Lomas et al., 1992). Polymers accumulate within the endoplasmic reticulum of hepatocytes in association with neonatal hepatitis, cirrhosis, and hepatocellular carcinoma. Concomitant deficiency of circulating α_1_-antitrypsin predisposes to severe, early onset emphysema (Gooptu and Lomas, 2008).

Previous data suggest that serpin polymers are formed by intermolecular linkage of the reactive center loop of one molecule with β sheet A of another (Dunstone et al., 2000; Gooptu et al., 2000; Huntington et al., 1999; Lomas et al., 1992; Schulze et al., 1990). However, a recent model suggests that the polymer is linked by a more extensive domain swap involving both the reactive center loop and strand 5 of β sheet A (Krishnan and Gierasch, 2011; Yamasaki et al., 2008). The controversy may be partly explained by our recent finding that structurally distinct polymers are favored by different conditions used to induce polymerization in vitro (Ekeowa et al., 2010). Resolving these issues at a residue-specific level is important for targeting drug design against the pathological polymerization of α_1_-antitrypsin deficiency. We have therefore used nuclear magnetic resonance (NMR) spectroscopy and a deficiency-associated and polymerogenic α_1_-antitrypsin variant to characterize structural and dynamic changes associated with population of the pathological intermediate state.

## Results

### Detection of α_1_-Antitrypsin_Queen's_

A mutation that, to our knowledge, has not previously been described was detected in an individual whose circulating α_1_-antitrypsin levels (median value 0.6 mg/ml; normal range 1.5–3.5 mg/ml) were between those expected of a Z homozygote and an MZ heterozygote. Genotyping revealed a compound heterozygote for the Z allele and a Lys154Asn variant that we have named α_1_-antitrypsin_Queen's_.

### Population of the Pathological Intermediate

Recombinant Lys154Asn α_1_-antitrypsin was characterized biochemically and biophysically (Figure S1, available online; Figures 1B and 1C). The variant had 64% of the functional activity of wild-type α_1_-antitrypsin (i.e., 1.6-fold greater stoichiometry of inhibition [SI]) with an apparent association rate constant (k_app'_) with bovine α-chymotrypsin of 5.5 (+/−1.1) × 10^4^ M^-1^s^-1^ (n = 3). When corrected for the increase in SI, the association rate constant (k_app_) for Lys154Asn α_1_-antitrypsin was 1.3 × 10^5^ M^-1^s^-1^, an order of magnitude lower than the wild-type control (1.16 × 10^6^ M^-1^s^-1^). Sodium dodecyl sulfate polyacrylamide gel electrophoresis (SDS-PAGE) confirmed increased substrate-like behavior in Lys154Asn α_1_-antitrypsin relative to the wild-type (Figure S1C).

The Queen's variant formed polymers under physiological conditions (Figure 1B). This was slow at 37°C, and more rapid at 42°C and higher temperatures. The general pathological relevance of these polymers was demonstrated by the presence of the 2C1 neo-epitope that is specific for polymers associated with disease (Miranda et al., 2010; Figure 1B, right).

Polymer formation occurs via population of an intermediate state (Dafforn et al., 1999). We therefore studied intermediate formation by Lys154Asn α_1_-antitrypsin. The variant populated intermediate states more readily than wild-type α_1_-antitrypsin in urea-induced and thermal denaturing conditions (Figure S1A; Figure 1C). Since we had established the disease relevance of the polymerogenic intermediate at temperatures in the physiological range, we focused on structural changes induced by incubation in these conditions. CD spectroscopy studies (Figures 1C and S1D) indicated that the mutant fold underwent a subtle structural transition between 35°C and 45°C before more dramatic transition between 50°C and 60°C. Increasing concentration did not affect the first transition but caused the second transition to commence at a lower temperature. Wild-type α_1_-antitrypsin remained stable to 60°C and underwent a sharp unfolding transition at higher temperatures, consistent with previous studies (Gooptu et al., 2009b; Parfrey et al., 2003). Increasing concentration cold-shifted the second half of the transition but did not affect its initial phase (Figure S1D). Increasing protein concentration 2.5-fold (0.4 to 1.0 mg/ml) reduced the midpoint of the overall transition for wild-type α_1_-antitrypsin by ∼1.5°C and that of Lys154Asn α_1_-antitrypsin by ∼1.0°C. The profiles of the first part of the transition observed for wild-type α_1_-antitrypsin and the first transition observed for Lys154Asn α_1_-antitrypsin are both concentration-independent. Therefore, this likely reports upon structural change within the molecule. The subsequent transition is concentration-dependent. It therefore likely reports, to some degree, upon polymerization of a species populated during the first phase.

Polymerogenic intermediate formation in α_1_-antitrypsin has been previously studied by ANS fluorescence. ANS fluoresces strongly upon binding to regions in which both polar and hydrophobic motifs are exposed to solvent and so binds to intermediate states, rather than fully folded or unfolded polypeptides. The ability of the Queen's variant of α_1_-antitrypsin to bind ANS was therefore assessed (Figure S1E). Lys154Asn α_1_-antitrypsin showed hyperfluorescence compared with the wild-type protein at 25°C. This was unexpected, since assays directly reporting structural information (CD spectroscopy, intrinsic fluorescence, and NMR spectroscopy) strongly indicated that both proteins were similarly well-folded and stable at this temperature. These observations of Lys154Asn α_1_-antitrypsin behavior are similar to those described for Z α_1_-antitrypsin (Knaupp et al., 2010). Moreover, incubating Lys154Asn α_1_-antitrypsin with ANS for longer resulted in even greater hyperfluorescence, whereas increasing temperature to 37°C did not enhance this further (Figure S1E). Wild-type α_1_-antitrypsin showed no change in ANS fluorescence at either temperature or upon prolonged incubation. These data indicate that for α_1_-antitrypsin variants in which conversion to the polymerogenic intermediate in solution is facile, ANS binding stabilizes the intermediate state sufficiently to skew the position of the native-intermediate equilibrium to the right. For Lys154Asn α_1_-antitrypsin, this effect is so significant that it outweighs any effect of temperature change between 25°C and 37°C. This supports our general view that the degree to which assays perturb solution equilibria must be considered when studying intermediate formation.

We hypothesized that ion-mobility mass spectrometry (IM-MS) might have sufficient sensitivity to detect population of the intermediate while minimally perturbing the solution equilibrium. At 20°C and 34°C, monomeric Lys154Asn α_1_-antitrypsin was indistinguishable from the wild-type protein by IM-MS. However, at 39°C, there was a 7.8% increase in the collision cross-section (CCS) of monomeric Lys154Asn α_1_-antitrypsin relative to the wild-type protein in keeping with population of an intermediate state (Figure 1C). The CCS of the intermediate state has been calculated as ∼18% greater than the native state, so a 7.8% increase in CCS is consistent with substantial population (∼40%) of the intermediate state in equilibrium at physiological temperatures.

### NMR Spectroscopic Characterization of α_1_-Antitrypsin Solution Behavior and Mutation Effects

**Figure 2:**
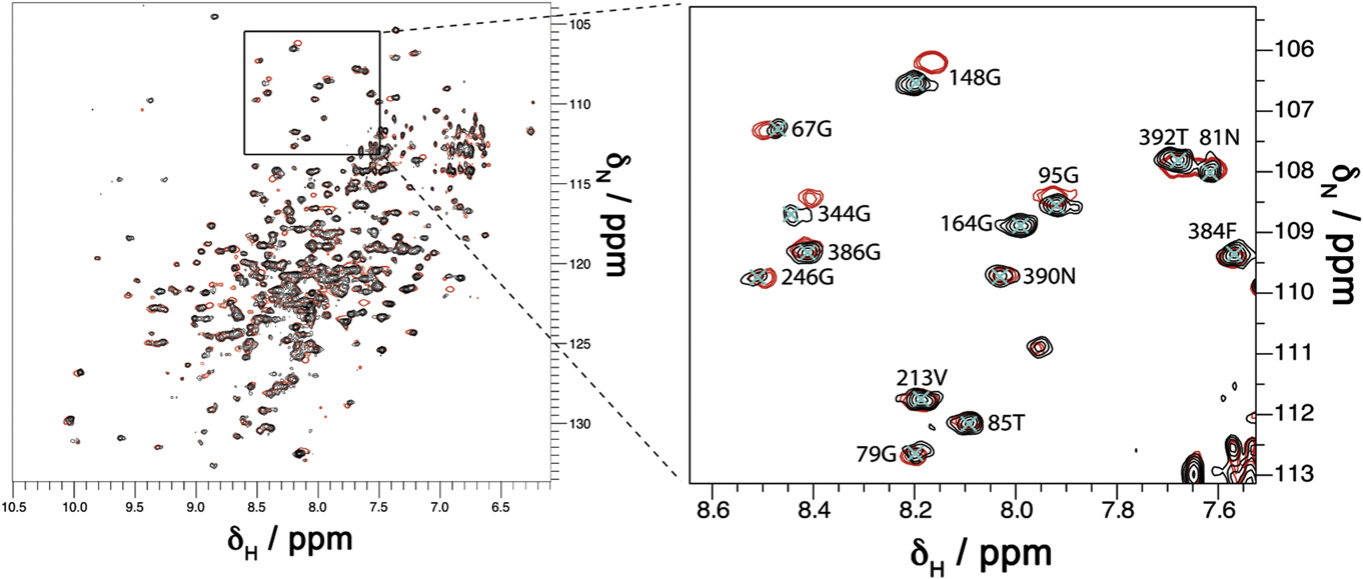
^1^H-^15^N TROSY-HSQC Spectrum of Wild-Type and Lys154Asn α_1_-Antitrypsin Zoom (right) illustrates examples of reporter residues (wild-type, black; Lys154Asn, red). See also Figure S2.

We have observed and assigned backbone resonances for almost all residues in wild-type α_1_-antitrypsin in a series of NMR experiments (Biological Magnetic Resonance Bank entry 17804; Nyon et al., 2011), allowing us to analyze its solution behavior (Figures 2 and S2). Chemical shift index analysis (Berjanskii and Wishart, 2006) was performed for Cα backbone bond signals alone or in conjunction with side-chain signals (Figure S2A). This method did not use any crystallographically derived predictions, so it permitted residue-specific comparison of the secondary structure behavior of the protein directly observed in solution with that observed crystallographically. Although most of the secondary structure behavior of individual residues was conserved between solution and the crystal lattice conditions, β sheet C showed considerable lability in solution. The solution behavior of helices C and I was reported as α-helical by Cα backbone bond signals. However, when side-chain signals were also considered, these reported random coil behavior. Such differences may indicate that these motifs also displayed some lability in solution (Figure S2A).

Two-dimensional (2D) NMR spectroscopy of ^15^N-labeled Lys154Asn α_1_-antitrypsin was then performed (Figure 2). At 25°C the well-dispersed regions of the spectrum contained a similar number (148) of cross-peaks to the number seen for the wild-type protein (152), and mean cross-peak intensities were similar for both proteins. A subset of 123 residues could be confidently assigned in the well-dispersed region of the ^1^H-^15^N TROSY-HSQC spectrum of Lys154Asn α_1_-antitrypsin. Overall peak integrals in the central region were also similar between the spectra for wild-type and Lys154Asn α_1_-antitrypsin (Figure S2B). Nevertheless intensity and chemical shift differences were induced by the presence of the mutation in the native state of the protein (Figure S2C). The degree of variation reported by intensity and chemical shift in the observed residues correlated closely. The magnitude of changes are shown by heatmap coloring of spheres mapped to the crystal structure and demonstrate the mutation most affects residues around the F-helix, strands 1–3 of β sheet A, and the reactive loop. In particular, the spectral effects were most dramatic on residues around hF and s3A, where the chemical shift changes were too great to allow their confident assignment in the mutant protein (Figure S2C, black spheres).

### NMR Spectral Changes and Polymerogenicity

**Figure 3:**
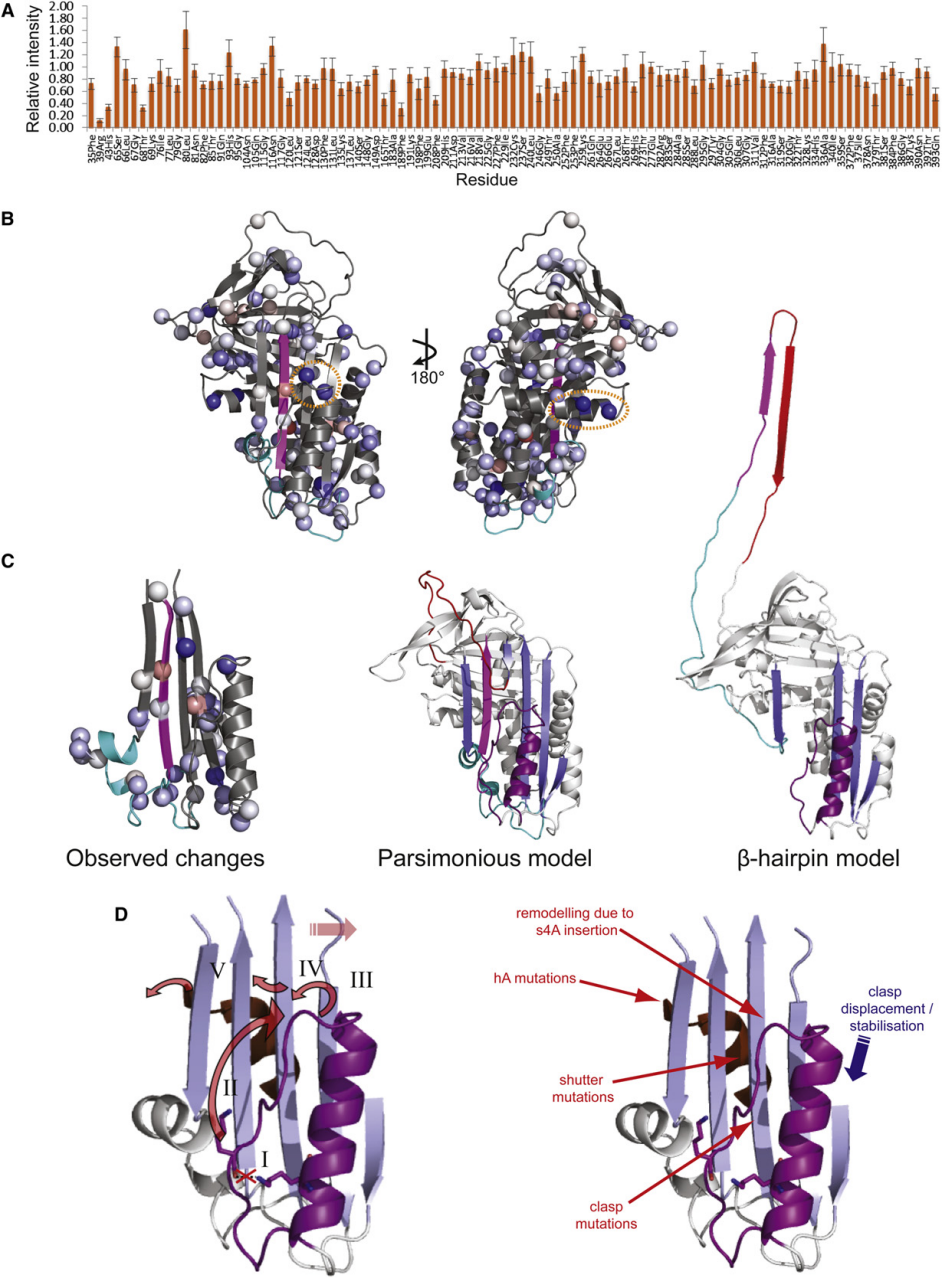
Population of the Intermediate by Lys154Asn α_1_-Antitrypsin under Physiological Conditions (A) Differential change in intensity for reporter cross-peaks in the ^1^H-^15^N TROSY-HSQC of Lys154Asn α_1_-antitrypsin at 37°C. Intensities are scaled relative to the wild-type protein at the same temperature and the intensities observed for Lys154Asn α_1_-antitrypsin relative to the wild-type at 25°C [(I_K154N,37_/I_wt,37_)/(I_K154N,25_/I_wt,25_)]. All intensities were scaled for concentration and 4,4-dimethyl-4-silapentane-1-sulfonic acid (DSS) control intensity. Error bars are defined according to variability observed in the datasets from which the values are derived, according to the formula$[\left(\frac{{\text{I}}_{\text{K}154\text{N},37}}{{\text{I}}_{\text{WT},37}}\right)/\left(\frac{{\text{I}}_{\text{K}154\text{N},25}}{{\text{I}}_{\text{WT},25}}\right)]\times \sqrt{{\left(\frac{{\text{d}}_{\text{K}154\text{N},37}}{{\text{I}}_{\text{K}154\text{N},37}}\right)}^{2}+{\left(\frac{{\text{d}}_{\text{WT},37}}{{\text{I}}_{\text{WT},37}}\right)}^{2}+{\left(\frac{{\text{d}}_{\text{K}154\text{N},25}}{{\text{I}}_{\text{K}154\text{N},25}}\right)}^{2}+{\left(\frac{{\text{d}}_{\text{WT},25}}{{\text{I}}_{\text{WT},25}}\right)}^{2}},$where I is the intensity for data obtained from wild-type (WT) or Lys154Asn (K154N) α_1_-antitrypsin at 25° or 37°C as indicated by the subscripts and d terms represent the observed standard deviation of peak intensities within each spectra. (B) Relative intensities quantified in (A) mapped onto the subset of reporter residues (spheres) in red-white-blue heatmap coloring (increases, red; unchanged, white; reductions, blue). Greatest change (maximally blue by RGB, residue 39) corresponds to a relative intensity of 0.11. Increasing redness indicates increasing intensity on the same scale. Ellipse: hA region reporting major change from native-like intensity. (C) (Left) Intensity change and stability observed in β sheet A, hF, hI, and connecting linkers. Current structural models of polymerogenic intermediates in the parsimonious (center) and β-hairpin (right) linkage models of polymerisation for comparison (β strand 5A magenta; hI and connecting linker cyan). (D) (Left) Unlocking the clasp. Proposed scheme of polymerogenic intermediate formation through propagation of conformational change from the mutation site through β sheet A (blue) and more distant motifs. The Lys154Asn mutation abolishes a hydrogen bond between hF and the neighboring linker (clasp, purple) region (I). Under physiological conditions this destabilization causes remodeling of the upper part of the clasp (II). Consequent structural changes in the upper part of s3A (III) facilitate lateral movement required to open the s4A site. Changes in s3A are also transmitted via (IV) the shutter region and s6B (brown) to the C-terminal residues of hA (V). Most of the residues within β sheet A remain within a native-like environment during formation of the intermediate. This is seen not only for residues in s5A but also for residues in hI and the connecting linker. (Right) Factors stabilizing (blue, arrow denotes stabilizing displacement of clasp region induced by the Gly117Phe mutation; Gooptu et al., 2009b) and destabilizing (red) the network of regulatory interactions. See also Figure S3.

Our principal aim was to assess changes in α_1_-antitrypsin associated with population of the disease-relevant intermediate state. Native state behavior was defined as that observed for wild-type α_1_-antitrypsin at the temperatures studied in which no polymerization was observed. At 37°C, slow, irreversible, and linear loss of the observable, monomeric sample due to polymerization was observed in Lys154Asn but not the wild-type α_1_-antitrypsin. The linearity indicated a constant rate of polymer formation and hence that the population of the polymerogenic intermediate was also constant, that is, the solution was in pseudo-equilibrium. ^1^H-^15^N TROSY-HSQC spectra reported residue-specific structural and dynamic changes in Lys154Asn α_1_-antitrypsin by nonuniform intensity change in assigned cross-peaks relative to wild-type α_1_-antitrypsin (Figure 3A). Values are scaled for monomer concentration; they take into account changes observed between wild-type and mutant protein at 25°C in the absence of polymerization and changes observed in wild-type α_1_-antitrypsin between 25°C and 37°C. They therefore report differential changes in Lys154Asn α_1_-antitrypsin associated with population of the polymerogenic state.

Most reporter cross-peaks signaled minimal change in Lys154Asn α_1_-antitrypsin at 37°C (Figure 3B). Those in strand 5 in β sheet A, helix I, and the connecting linker strongly preserved intensity (Figure 3C). NMR intensity data are highly sensitive reporters of changes in structure and dynamics, so relatively large changes may be induced by quite small changes in solution behavior. Nevertheless, residues reporting the most dramatic intensity change in the polymerogenic ensemble notably occur in regions previously linked with conformational change (upper s3A, upper hF, underlying the upper s4A site, and hA; Cabrita et al., 2004; Mahadeva et al., 1999; Zhou et al., 2003).

A further 12 distinct cross-peaks (signal:noise 11.5) were observed in the well-dispersed region of the spectrum in Lys154Asn but not the wild-type α_1_-antitrypsin at 37°C (Figure S3A). The new cross-peaks disappeared on cooling and so may represent residues within a nonnative monomeric state, rather than polymers, since these did not dissociate (and would predictably give peaks of very low intensity). If so, these data support a process of native-non-native monomeric conformational exchange that is slow (≥ms) by NMR timescales, consistent with previous data (Dafforn et al., 1999).

The mutation will mildly perturb local electrostatic interactions involving Lys154 (Figure S3B). Its effects on resonances of nearby residues were too great to dissect by cross-peak analysis. We therefore studied a eukaryotic cell model of disease (Gooptu et al., 2009b) by mutagenesis. These clarified that the hydrogen bond of Lys154 (F-helix) with the main-chain carbonyl oxygen of Lys174 (neighboring linker) regulated against polymer formation (Figure S3B).

The residue-specific changes caused by the Lys154Asn mutation differed markedly from those observed in urea conditions causing a similar degree of polymerization (1.5 M; Figure S3C). Both patterns differ from that indicated by studies in guanidine or low pH conditions (Krishnan and Gierasch, 2011; Yamasaki et al., 2008). Thus, the polymerogenic monomer ensemble to which this mild deficiency mutation reduces the energetic barrier, differs structurally and/or dynamically from polymerogenic ensembles induced by other conditions. The resultant polymers are also structurally distinct (Ekeowa et al., 2010).

## Discussion

Studying key native to intermediate transitions can elucidate mechanisms of the conformational diseases and aid targeting of novel therapeutic strategies. However, the intrinsic tendency of intermediates to adopt more stable, polydisperse, and multimeric conformations renders this inherently challenging. Moreover, structurally distinct serpin polymers are induced by chemical denaturants compared to pathological mutations or heating (Ekeowa et al., 2010). Techniques that can perturb equilibria by stabilizing particular structural endpoints (e.g., selective labeling, proteolysis, and/or peptide binding) may also skew characterization of intermediate ensembles in conformational diseases. We have therefore undertaken the residue-specific study of a *forme fruste* disease mutant in solution, under physiological conditions, using minimally perturbing techniques. This is arguably the best strategy for studying intermediate states relevant to conformational diseases.

Lys154Asn α_1_-antitrypsin populates a polymerogenic intermediate state at 37°C. Polymers formed from Lys154 mutants recapitulate the pathognomic 2C1 neo-epitope. Polymerization of Lys154Asn α_1_-antitrypsin is therefore a good structural model for the pathological polymerization in vivo of more common and severe variants, such as Z α_1_-antitrypsin. Serendipitously, polymerization occurs slowly enough to allow the study of the solution behavior of α_1_-antitrypsin when the pathological intermediate state is populated. We have overcome obstacles posed by the molecule's large size (45 kDa) to use NMR spectroscopy and characterize residues that remain native-like or alter their solution behavior in association with formation of the pathological intermediate. Our data are consistent with sufficient population of the intermediate state in Lys154Asn α_1_-antitrypsin at physiological temperatures to contribute to our observations. Most observed residues behave in a highly native-like way under these conditions. Relative intensity values indicate preservation of native-like signal after correcting for changes due to the mutation at 25°C and changes in protein tumbling and polymerization at 37°C. A value of 100% would therefore indicate entirely native-like structural and dynamic behavior in a particular residue. The mean relative intensity across the entire subset of residues that could be analyzed in this way was 82%. It is therefore unlikely that the intermediate ensemble is substantially unfolded as required for extensively domain-swapped models of polymerization. Specifically, analysis of cross-peak intensities supports high stability in the hI-s5A region proposed to unfold in the β-hairpin model (Figure 3C). This is indicated by relative intensities of 90% for the seven residues within this region compared with the equivalent cross-peaks in the native state. Similar preservation of native-like signal is indicated for the core strands 4 and 5 of β sheet B (residues 370–388).

Larger changes (Figures 3A and 3B) may be due to a number of differences in structural and/or dynamic behavior, so their interpretation is complex. Nevertheless, the localization of the greatest changes from wild-type native behavior is striking. Such changes are reported by residues in regions associated with remodeling during formation of the intermediate state in parsimonious models of polymerization (Figure 3B, left panel, circled: Thr165 at the top of hF, relative intensity 0.46, Phe189, upper s4a, 0.31). The effects on these residues cannot be simply explained by proximity to the mutation site, since they lie further from it than many residues that report highly native-like behavior. Residues reporting a major loss of relative intensities are also observed at the C-terminal end of hA (Figure 3B, right panel ellipse: Arg39, relative intensity 0.11 and His 43, 0.33), even further from the mutation site. This corresponds to the site of another polymerogenic α_1_-antitrypsin deficiency mutation (Ile39Cys; Mahadeva et al., 1999). Mutations in nearby serpin residues (Tyr38Ser, antithrombin; Leu41Pro, α_1_-antitrypsin) are also associated with deficiency and disease (Dafforn, 1999). Our data suggest that structural and/or dynamic changes here are associated with formation of the pathological intermediate. The effects would be transmitted most directly to the A-helix from s3A via the intervening shutter and s6B motif (Figure 3D), known hotspots for pathological, polymerogenic mutations.

Taking together the data from biochemical, biophysical, enzyme-linked immunosorbent assay (ELISA) and cell studies, and previous observations (Bruce et al., 1994; Sharp et al., 1999), we propose that the helix F and linker region constitutes a “clasp” motif (Figure 3D, purple). This may seal a network of interactions that regulate conformational change. Compromise of the network is associated with formation of the pathological intermediate.

Our data indicate that the methods used to induce intermediate formation when studying conformational diseases can be major determinants of the structural distribution of intermediate states within the solution ensemble. A recent study undertook residue-specific labeling of single Cys mutants during early guanidine denaturation. It supported extensive unfolding of strands 5 and 6, as well as helices F and I (Krishnan and Gierasch, 2011). That pattern differs from our observations, both in the presence of the Lys154Asn mutation at 37°C and in low concentrations of urea. The modulation of monomeric ensembles to produce analogous but structurally distinct polymer states is relevant to studies of other conformational diseases, where chemical denaturants are commonly used in vitro to induce multimerization. It may also explain the phenomenon of strain specificity observed in conformational disorders, such as prion diseases and amyloidoses.

We have demonstrated that NMR spectroscopy is optimal for studying pathological intermediate formation in the serpinopathies, providing residue-specific detail without perturbing dynamic solution equilibria. Comparison of wild-type α_1_-antitrypsin characteristics with those of mild deficiency variants also powerfully dissects disease-relevant behavior. Such studies appear timely, given the range of proposed polymerisation models based upon data obtained using a range of more perturbing techniques. Further studies using different variants and NMR modalities, for example, detailed dynamics studies, will further characterize this contentious mechanism of pathological conformational change to optimize drug design and so treat the serpinopathies.

## Experimental Procedures

### Molecular Biology, Protein Purification, and Cell-Free Characterization

Mutations were introduced into α_1_-antitrypsin cDNA within the pQE31 and/or the pcDNA3.1 plasmids by polymerase chain reaction mutagenesis (Zhou et al., 2001; Gooptu et al., 2009b). pQE31 plasmids containing cDNA encoding hexahistidine-tagged, recombinant α_1_-antitrypsin were transfected into XL1 Blue *Escherichia coli* (Stratagene, Santa Clara, California, USA). The proteins were expressed and purified as previously described (Parfrey et al., 2003). They were characterized using SDS-, nondenaturing and transverse urea gradient (TUG)-PAGE, circular dichroism (CD), intrinsic and 8-anilinonaphthalene-1-sulfonate (ANS) fluorescence spectrometry and by enzyme inhibitory activity and kinetics assays (Dafforn et al., 1999, 2004; James et al., 1999; Stone and Hofsteenge, 1986).

### Ion Mobility Mass Spectrometry

Samples were buffer exchanged into 100 mM ammonium acetate (pH 7.0), desalted, and concentrated to a final concentration of 20 μM. Proteins were introduced to the mass spectrometer by nano electrospray ionization. All IM-MS experiments were performed in a hybrid quadrupole, orthogonal acceleration time-of-flight (oa-TOF) mass spectrometer equipped with a traveling wave (T-Wave) ion mobility separation device (Synapt HDMS, Waters, Manchester, UK; Pringle et al., 2007). The source temperature was 40°C, capillary voltage optimized 1.0–1.2 kV, and cone voltage was 20 V. The pressure in the T-Wave ion mobility cell was 0.55 mbar. The mobility gas was nitrogen. T-Wave height and velocity were set at 10 V and 300 m/s, respectively. Arrival time distributions were converted to collision cross-sections (CCS) by power fit (Hilton et al., 2010; Thalassinos et al., 2009). The calibrant protein was equine myoglobin (Sigma-Aldrich, Gillingham, UK). Data acquisition and processing were carried out using MassLynx 4.1 software (Waters Corp., Milford, MA, USA). Data are the mean (+/− SD) of three repeats.

### Sample Preparation for NMR Spectroscopy

cDNA coding for hexahistidine-tagged α_1_-antitrypsin within a pQE31 plasmid was transformed into strain BL21-Gold (DE3) *E. coli* (Stratagene). Cells were cultured in M9 minimal media (in H_2_O or D_2_O) at 37°C with 1 g/l of ^15^NH_4_Cl (Spectra Stable Isotopes, Columbia, MD, USA) and 2 g/l of either glucose or ^13^C-glucose (Sigma-Aldrich) as the sole nitrogen and carbon sources, respectively. Following induction of protein expression cells were grown for 6 hr, rather than the 4 hr, period used with rich media. In all other respects protein expression and purification was carried out as described above. Samples were stored in 25 mM Na_2_HPO_4_ (pH 8.0), 50 mM NaCl, and 1 mM ethylenediaminetetraacetic acid. Sample homogeneity was confirmed by SDS-, nondenaturing and transverse urea gradient (TUG)-PAGE and by assessment of inhibitory activity.

### NMR Spectroscopy Conditions

NMR spectra used to compare wild-type and Lys154Asn α_1_-antitrypsin were collected on uniformly ^15^N-labeled samples at 298 K, 175 μM on a Varian UnityInova 600 MHz spectrometer with an HCN cryoprobe. Ten percent D_2_O/1% 4,4-dimethyl-4-silapentane-1-sulfonic acid (DSS) was added to concentrated α_1_-antitrypsin samples prior to NMR spectroscopy.

Backbone assignments were obtained from TROSY (Pervushin et al., 1997) versions of HNCO, HN(CA)CO, HNCA, HN(CO)CA, HNCACB, and HN(CO)CACB spectra (Eletsky et al., 2001; Salzmann et al., 1998), together with a 3D NOESY-TROSY-^15^N HSQC spectrum (Zhu et al., 1999). ^2^H decoupling (1 kHz WALTZ-16; Shaka et al., 1983) was applied in triple-resonance experiments. Magnetization was transverse on CA or CB. Two-dimensional TROSY-^15^N HSQC spectra for assignment. Wild-type and Lys154Asn α_1_-antitrypsin comparison spectra were recorded using single-transition-to-single-transition polarization transfer and phase cycling for coherence order selection (Rance et al., 1999; Zhu et al., 1999). Spectra were processed and analyzed using nmrPipe (Delaglio et al., 1995) and CCPN (Fogh et al., 2002). All spectra were referenced to DSS at 0.0 ppm, manually phased and baseline corrected. Lys154Asn α_1_-antitrypsin spectra were analyzed by changes in cross-peak intensity relative to the data on wild-type α_1_-antitrypsin (I_K154N_/I_wild-type_) and magnitude of chemical shift change (Δδ). This was calculated as

$$\sqrt{{\left(\Delta \delta^{1}\text{H}\right)}^{2}+{\left(\Delta \delta^{15}\text{N}/5\right)}^{2}},$$

where Δ^1^H was the change in the chemical shift along the ^1^H axis andΔ^15^N the change in chemical shift along the ^15^N axis (parts per million [ppm]).

### Cell Biological Characterization

The pcDNA3.1 plasmids containing wild-type and mutant α_1_-antitrypsins were transiently transfected into COS-7 cells. The resulting protein expression was characterized by western blot analyses of SDS- and nondenaturing PAGE of intracellular material as previously described (Miranda et al., 2008, 2004). Luciferase controls were used to confirm equivalent loading between samples. The polymer load was quantified by ELISA using the polymer-specific 2C1 monoclonal antibody as the primary antibody (Miranda et al., 2010).
