# Supplementary material for: Structural Dynamics Associated with Intermediate Formation in an Archetypal Conformational Disease
Source: Structure. 2012 Mar 7;20-135(3-3):504–12. doi: 10.1016/j.str.2012.01.012 (PMC3314904; doi:10.1016/j.str.2012.01.012)
Supplement: Document S1. Figures S1–S3 [file mmc1.pdf]

Structure, Volume *20*

## Supplemental Information

# Structural Dynamics Associated with Intermediate Formation in an Archetypal Conformational Disease

**Mun Peak Nyon, Lakshmi Segu, Lisa D. Cabrita, Géraldine R. Lévy, John Kirkpatrick, Benoit D. Roussel, Anathe O.M. Patschull, Tracey E. Barrett, Ugo I. Ekeowa, Richard Kerr, Christopher A. Waudby, Noor Kalsheker, Marian Hill, Konstantinos Thalassinos, David A. Lomas, John Christodoulou, and Bibek Gooptu**

**Inventory of Supplemental Information**

**Fig. S1**
**Biochemical and biophysical characterisation of Lys154Asn $\alpha_1$-antitrypsin compared to the wild-type protein. This accompanies Fig. 1.**

**Fig. S2**
**Solution behaviour of native wild-type and Lys154Asn $\alpha_1$-antitrypsin reported by NMR spectroscopy (chemical shift analysis and comparison of $^1H$-$^{15}N$ TROSY HSQC data). This accompanies Fig. 2.**

**Fig. S3**
**NMR spectroscopic studies of observed changes associated with intermediate formation and mutagenesis studies of local mediation of the mutation's effects in Lys154Asn $\alpha_1$-antitrypsin. This accompanies Fig. 3.**

## Supplementary Figures

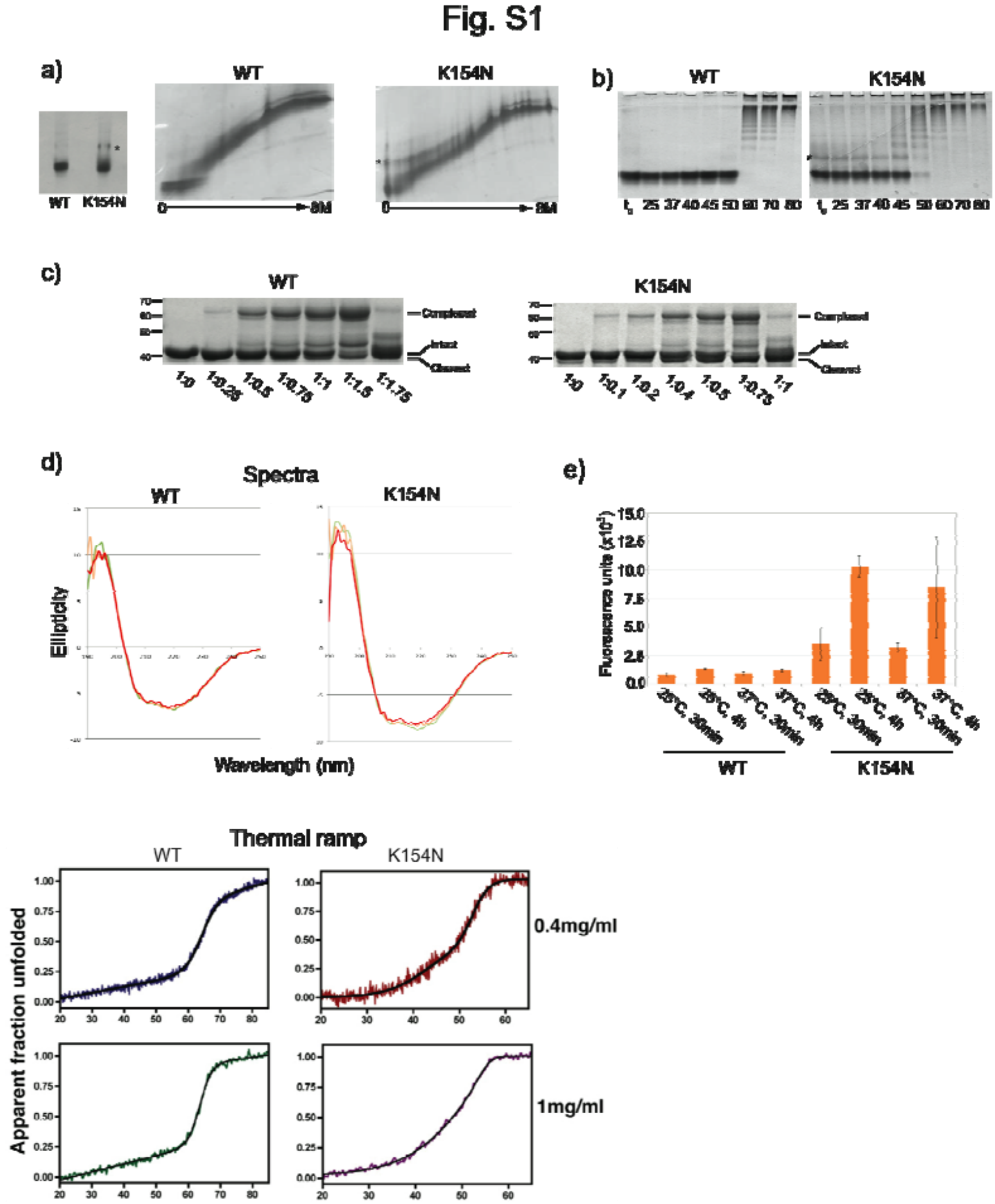

# Fig. S2

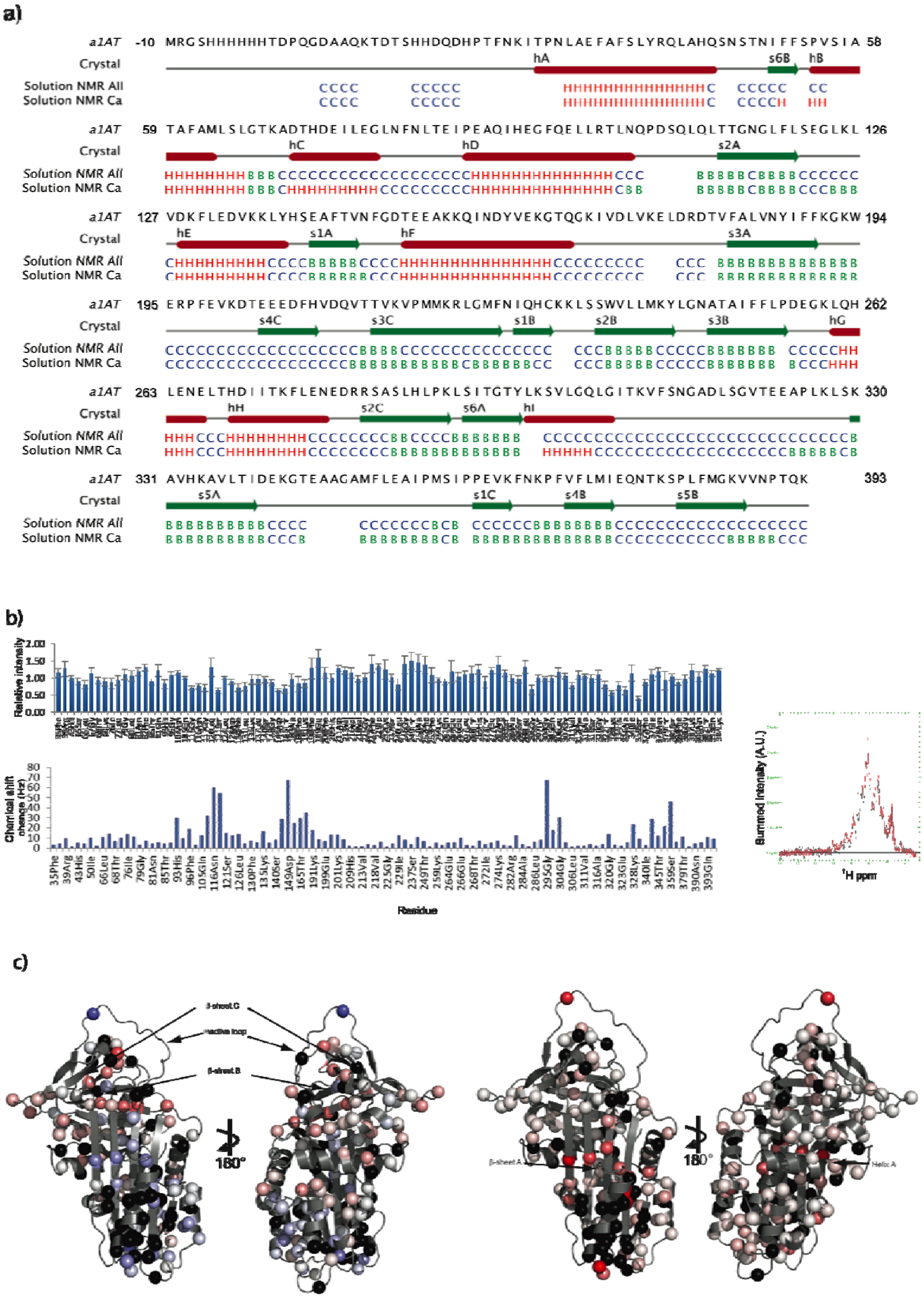

a)
a1AT
Crystal
Solution NMR All
Solution NMR Ca
hA
hB
hC
hD
hE
hF
hG
hH
hI
s1A
s2A
s3A
s4C
s3C
s1B
s2B
s3B
s2C
s6A
s5A
s1C
s4B
s5B
s6B
b)
Relative intensity
Chemical shift change (Hz)
Residue
Summed Intensity (A.U.)
$^{1}$H ppm
c)
β-sheet C
reactive loop
β-sheet B
180°
β-sheet A
Helix A

# Fig. S3

a)

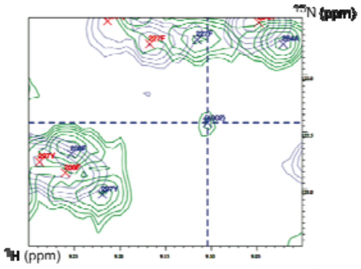
15N (ppm)
1H (ppm)

b)

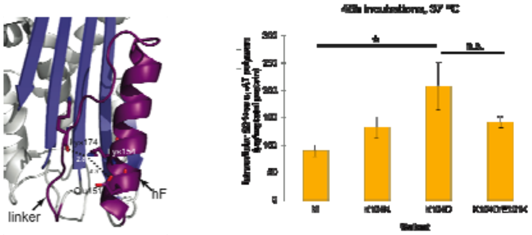
linker
hF
48h incubations, 37 °C
*
n.s.

c)

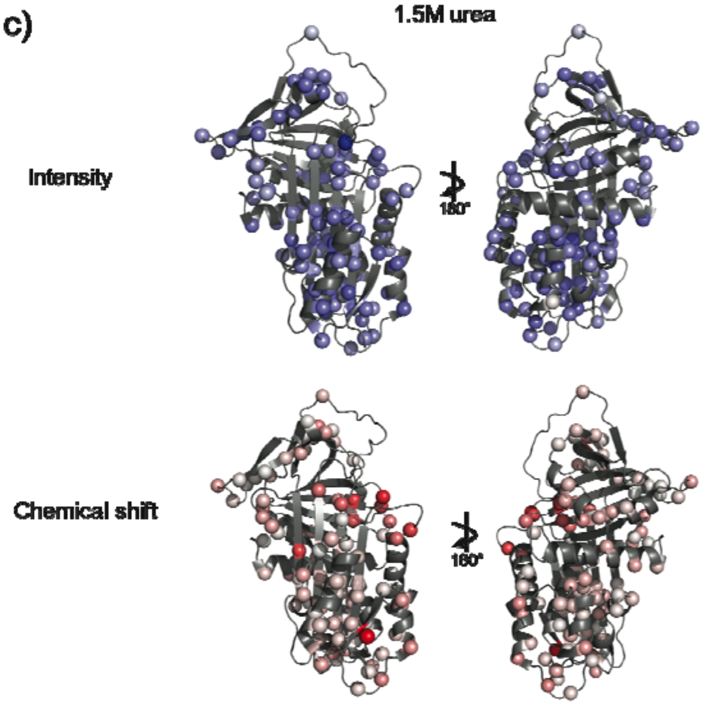
1.5M urea
Intensity
180°
Chemical shift
180°

**Supplementary Figure Legends**

**Figure S1. Biochemical and biophysical characterisation of Lys154Asn $\alpha_1$-antitrypsin (related to Figure 1)**

a) 7.5% (w/v) native PAGE (left panel) of Lys154Asn $\alpha_1$-antitrypsin (K154N) compared with wild-type (WT) control; * marks migration of an intermediate. Right panel, 0-8M urea transverse urea gradient (TUG)-PAGE.

b) 7.5% (w/v) native PAGE of wild-type (left) and Lys154Asn (right) $\alpha_1$-antitrypsin incubated (1 mg/ml) at a range of temperatures for 30 minutes. Lanes contain 8 μg of protein. Incubations were performed at pH 7.4. Identical temperature-dependent polymerisation was observed at pH 7.0 and 8.0.

c) 10% SDS-PAGE of wild-type (WT, left) and Lys154Asn (K154N, right) $\alpha_1$-antitrypsin interactions with bovine α-chymotrypsin. Molecular weights (kDa) are indicated by marker bars. Molar ratios of $\alpha_1$-antitrypsin:chymotrypsin are shown beneath each lane.

d) CD spectroscopy of wild-type (WT) and Lys154Asn (K154N) $\alpha_1$-antitrypsin. Upper panel, far u-v spectra (10 spectra acquired at each temperature point, ellipticity measured in mdeg). Lower panel, comparison of thermal ramp behaviour at 2 different concentrations (0.4 mg/ml (same data as in Fig. 1c), and 1.0 mg/ml (n=3)).

e) Effects of mutation, temperature and incubation time of wild-type (WT) and Lys154Asn (K154N) $\alpha_1$-antitrypsin with ANS upon fluorescence at 480 nm wavelength (n=3 for each condition, error bars represent standard deviation values).

**Figure S2. Solution behaviour and residue-specific changes induced by the Lys154Asn mutation in $\alpha_1$-antitrypsin (related to Figure 2)**

a) NMR spectral characterization of native $\alpha_1$-antitrypsin allows data on solution behaviour to be related to crystallographically defined secondary structure of α-helices (red lines) and β-strands (green arrows) is highlighted and labelled. Below this the solution secondary structure behaviour is indicated, based upon direct analysis of chemical shifts of the assigned cross-peaks in NMR spectra. These are reported for mainchain Cα nuclei alone (Ca), or including sidehain atom behaviour (all). C = random coil, H = α-helix, B = β-strand. In most cases the solution secondary structure behaviour reported in solution matched that seen crystallographically. However residues in 3 motifs reported increased lability in solution. There was more random coil-like structure in β-sheet C and to a lesser degree, in helices C and I. Unsurprisingly, a few end turns of α-helices (e.g. hD, hE, hF) also reported less helical characteristics in solution. Data from the residues beyond the crystallographic C-termini of hB and s3A, key regulators of β-sheet A expansion and polymerisation, indicated extension of these motifs in solution. Reactive loop and s1C residues indicated similar β-strand characteristics for the mainchain but random-coil like characteristics for sidechain data.

b) Left; Changes in cross-peak intensity (above) and magnitude of cross-peak chemical shift (Δδ, below) for Lys154Asn relative to wild-type $\alpha_1$-antitrypsin at 25 C for the subset of unambiguous resonances in 2D spectra. Right; $^1$H dimension integral of cross-peaks in the central, overlapped region of the spectra for wild-type (black) and Lys154Asn (red) $\alpha_1$-antitrypsin. Error bars are defined according to the standard deviation (d) observed for peak intensities (I) across the wild-type (WT) or Lys154Asn (K154N) datasets at 25°C using the formula:

$$[(I_{K154N}/I_{WT})] \times \sqrt{\left(\frac{d_{K154N}}{I_{K154N}}\right)^2 + \left(\frac{d_{WT}}{I_{WT}}\right)^2}$$

c) Intensity (left) and chemical shift (right) data shown in b) plotted on the subset of followed residues (spheres) within the structure of $\alpha_1$-antitrypsin. Red-white-blue heatmap colouring is

used (increases - red, no change – white, decreases – blue ). For the relative intensities the greatest change was an increase in relative intensity to 1.58 (residue 199): represented by maximal redness on an RGB colour scale. Reductions in relative intensity are represented by increasing blueness according to the same scale. Residues corresponding to cross-peaks that could not be followed from the $^{1}H$-$^{15}N$ TROSY-HSQC spectrum of wild-type $\alpha_1$-antitrypsin at 25°C to the equivalent spectrum for Lys154Asn are shown in black. Other residues are coloured grey. For chemical shift data all changes were calculated as magnitudes and were therefore positive. The maximum change that could be assigned to a specific residue (0.169 ppm), occurred at residue 149 and so this is represented by maximal RGB scale redness.

**Figure S3 (related to Figure 3)**

a) $^{1}H$-$^{15}N$ TROSY-HSQC of Lys154Asn $\alpha_1$-antitrypsin at 25°C (grey contours, blue labels) and 37°C (green contours, red labels). Example of an 'extra' cross-peak appearing at 37°C in Lys154Asn $\alpha_1$-antitrypsin but not seen at 25°C or in wild-type $\alpha_1$-antitrypsin at either temperature is highlighted (dashed intersection).

b) Left; The Lys154 sidechain participates in 2 polar interactions, a salt bridge to Glu151 nearby on hF and a hydrogen bond to the mainchain carbonyl of Lys174. Right; Probing the mechanism of conformational dysregulation in Lys154Asn $\alpha_1$-antitrypsin by mutagenesis in a mammalian cell model (Gooptu et al., 2009). Pathological polymer load within cells quantified by 2C1 mAb ELISA (Miranda et al., 2010) (calibrated using Z $\alpha_1$-antitrypsin polymers; values are mean +/- SD of 3 repeats, * indicates $p<0.05$, n.s. non-significant). Local polar interactions are mildly disturbed by the Lys154Asn mutation, the small increase in intracellular polymer accumulation is consistent with the minor clinical effect on circulating levels. This effect can be enhanced by making the more severe Lys154Asp

mutation, as reported by increased intracellular polymer load. It is not possible to avoid a mainchain carbonyl at residue 174. Therefore to dissect the relative effects of perturbing the 2 polar interactions we instead focused upon the salt bridge to Glu151. In the context of the Lys154Asp mutation, restoring the potential for salt bridging by mutating Glu151 to a lysine (K154D/E151K) did not significantly rescue the phenotype. These data indicate that the remaining polar interaction of Lys154, a sidechain-mainchain hydrogen bond between hF and the neighbouring linker, is a regulator of polymerogenic conformational change.

c) Residue-specific changes in intensities (upper) and chemical shifts (lower) reported when a polymerogenic intermediate ensemble in wild-type $\alpha_1$-antitrypsin is accessed at low (1.5 M) concentrations of urea. The changes are clearly different to those observed under non-denaturant, physiological conditions as a result of the Lys154Asn deficiency mutation. Thus lowering the energetic barrier to intermediate formation in $\alpha_1$-antitrypsin due to the presence of a pathological mutation generates a distinct intermediate ensemble to that induced by denaturant even when both ensembles are polymerogenic. This parallels the distinction between $\alpha_1$-antitrypsin polymers formed by denaturant and those formed by heating or due to pathological mutations.
